# Associations between essential medicines and health outcomes for cardiovascular disease

**DOI:** 10.1186/s12872-021-01955-1

**Published:** 2021-03-25

**Authors:** Liane Steiner, Shawn Fraser, Darshanand Maraj, Nav Persaud

**Affiliations:** 1 MAP Centre for Urban Health Solutions, St. Michael’s Hospital, Toronto, ON Canada; 2 Athabasca University, Athabasca, AB Canada; 3 Department of Family and Community Medicine, University of Toronto, Toronto, ON Canada

**Keywords:** Cardiovascular disease, Essential medicines, Amenable mortality

## Abstract

**Background:**

National essential medicines lists are used to guide medicine reimbursement and public sector medicine procurement for many countries therefore medicine listings may impact health outcomes.

**Methods:**

Countries’ national essential medicines lists were scored on whether they listed proven medicines for ischemic heart disease, cerebrovascular disease and hypertensive heart disease. In this cross sectional study linear regression was used to measure the association between countries’ medicine coverage scores and healthcare access and quality scores.

**Results:**

There was an association between healthcare access and quality scores and health expenditure for ischemic heart disease (p ≤ 0.001), cerebrovascular disease (p ≤ 0.001) and hypertensive heart disease (p ≤ 0.001). However, there was no association between medicine coverage scores and healthcare access and quality scores for ischemic heart disease (p = 0.252), cerebrovascular disease (p = 0.194) and hypertensive heart disease (p = 0.209) when country characteristics were accounted for.

**Conclusions:**

Listing more medicines on national essential medicines lists may only be one factor in reducing mortality from cardiovascular disease and improving healthcare access and quality scores.

**Supplementary Information:**

The online version contains supplementary material available at 10.1186/s12872-021-01955-1.

## Introduction

Approximately 29% of deaths worldwide are from cardiovascular disease specifically, ischemic heart disease, stroke and hypertensive heart disease [1]. The burden of these and other non-communicable diseases (NCD) will be associated with productivity loss and catastrophic healthcare costs [2] which has the potential to significantly undermine national macroeconomic development [3]. Deaths from cardiovascular disease are amenable to healthcare including treatments such as antihypertensives [4].

Following a 2011 United Nations meeting, the World Health Organization (WHO) released a briefing document which stated that the burden of NCD’s cannot be reduced without access to essential medicines [5]. Essential medicines are those that satisfy the priority health care needs of the population [6]. The purpose of an essential medicines list is to ensure quality medicines are available in a functioning health system, in appropriate forms, at affordable prices for both the individual and the community [6]. The WHO created a Model List of Essential Medicines (WHO Model List) which provides recommendations for minimum medicine needs for a basic health-care system. More than 100 countries have embraced the idea of essential medicines and adapted their own national essential medicines list (NEML) to address their health care priorities informed by their national burden of disease [2]. NEMLs are used to guide appropriate use of medicines, as well as medicine selection, reimbursement and public sector procurement [7, 8]. In the public sector, essential medicines are more available than other medicines, suggesting that there may be preferential attention from governments given to them, therefore carefully selecting and adopting an NEML is the first step in ensuring equitable access to pharmaceutical treatment [2]. Medication availability and accessibility plays and important role in addressing the burden of NCD’s [3] as evident by a reduction in mortality and morbidity in many countries since the implementation of essential medicines [9]. Population mortality that is amenable to care is assessed by the healthcare access and quality (HAQ) score that is available for 195 countries and that is comprised of 32 causes of death including ischemic heart disease, cerebrovascular disease and hypertensive heart disease [4].

The purpose of this study was to determine the relationship between listing essential medicines used to treat ischemic heart disease, cerebrovascular disease and hypertensive heart disease and amenable mortality related to these conditions measured by the HAQ score [4].

## Methods

### Dataset sources

All medications, with some exceptions, from countries’ NEMLs hosted in the WHO’s National Essential Medicines Lists Repository were extracted and recorded in an Excel database [10, 11]. NEMLs for 137 countries were identified [10].

We used the 2018 amenable mortality subscores, calculated by measuring age standardized mortality rates, for ischemic heart disease, cerebrovascular disease and hypertensive heart disease [4].

### Inclusion criteria

Countries were included if they had a NEML captured by the Global Essential Medicines (GEM) database and a HAQ score for ischemic heart disease, cerebrovascular disease and hypertensive heart disease.

### Data collection

In order to identify which medications were relevant to the three causes of interest (ischemic heart disease, cerebrovascular disease and hypertensive heart disease), we searched for guidelines for ischemic heart disease, cerebrovascular disease and hypertensive heart disease on the WHO website in June 2019. Four international guidelines distributed by the WHO, an internationally recognized health authority, were selected: Prevention and Control of Non-communicable Diseases: Guidelines for primary health care in low-resource settings [12], WHO Package of Essential Non-communicable Diseases Interventions for Primary Health Care in Low-Resource Settings [13], Technical Package for cardiovascular disease management in primary health care- evidence-based treatment protocols [7], Tackling NCDs: “Best Buys” and other recommended interventions for the prevention and control of non-communicable diseases [14]. Although it is not an internationally recognized guideline, additional guidance from the American Heart Association’s website was used to ensure all relevant medicines were captured [15]. These guidelines along with the WHO Model List 20th edition [16] were used to identify medicines used for treatment of ischemic heart disease, cerebrovascular disease and hypertensive heart disease. Guidelines were searched using the causes and associated International Classification of Diseases 10th revision codes provided by the HAQ score [4].

Population size, health expenditure and life expectancy were retrieved from the Global Health Observatory [17]; prevalence for ischemic heart disease, cerebrovascular disease and hypertensive heart disease was retrieved from the Global Burden of Disease Study [1]. Most data was for the year 2016; if 2016 data was not available, data from the closest year to 2016 was retrieved. Country characteristics can be found in Table 1.

**Table 1:** Country Characteristics

Country	ISO code	Geographic region	Income group	Ischemic heart disease medicine coverage score	Cerebrovascular disease medicine coverage score	Hypertensive hear disease medicine coverage score	Health expenditure for 2015 (per capita in PPP intl$)	Population for 2016 (in thousands)	Life expectancy for 2016 (in years)	Year of NEML publication
Afghanistan	AFG	Eastern Mediterranean	Low	22	15	14	183.9	34,656	62.7	2014
Albania	ALB	Europe	Upper middle	34	30	26	773.7	2926	76.4	2011
Algeria	DZA	Africa	Upper middle	47	44	36	1031.2	40,606	76.4	2016
Angola	AGO	Africa	Lower middle	5	3	2	195.5	28,813	62.6	2007
Antigua and Barbuda	ATG	The Americas	High	28	21	17	1105.1	101	75	2008
Argentina	ARG	The Americas	High	35	27	21	1389.8	43,847	76.9	2011
Armenia	ARM	Europe	Upper middle	24	17	13	883.2	2925	74.8	2010
Bahrain (Kingdom of)	BHR	Eastern Mediterranean	High	37	31	26	2453.2	1425	79.1	2015
Bangladesh	BGD	South-East Asia	Lower middle	19	12	10	88	162,952	72.7	2008
Barbados	BRB	The Americas	High	57	51	42	1233.6	285	75.6	2011
Belarus	BLR	Europe	Upper middle	33	28	21	1084.6	9480	74.2	2012
Belize	BLZ	The Americas	Upper middle	30	23	20	523.7	367	70.5	2008
Bhutan	BTN	South-East Asia	Lower middle	30	23	18	287.1	798	70.6	2016
Bolivia	BOL	The Americas	Lower middle	28	20	16	445.8	10,888	71.5	2011
Bosnia and Herzegovina	BIH	Europe	Upper middle	28	24	19	1101.8	3517	77.3	2009
Botswana	BWA	Africa	Upper middle	28	22	17	970	2250	66.1	2012
Brazil	BRA	The Americas	Upper middle	31	24	18	1391.5	207,653	75.1	2014
Bulgaria	BGR	Europe	Upper middle	55	53	45	1491.9	7131	74.8	2011
Burkina Faso	BFA	Africa	Low	24	17	13	96.1	18,646	60.3	2014
Burundi	BDI	Africa	Low	24	18	13	63.7	10,524	60.1	2012
Cabo (Cape) Verde	CPV	Africa	Lower middle	42	35	28	310.4	540	73.2	2009
Cambodia	KHM	South-East Asia	Lower middle	2	1	0	209.6	15,762	69.4	2003
Cameroon	CMR	Africa	Lower middle	27	20	14	162.8	23,439	58.1	2010
Central African Republic	CAF	Africa	Low	25	19	14	31.9	4595	53	2009
Chad	TCD	Africa	Low	17	12	9	99.8	14,453	54.3	2007
Chile	CHL	The Americas	High	28	21	18	1903.1	17,910	79.5	2005
China	CHN	Western Pacific	Upper middle	30	25	19	762.2	1,411,415	76.4	2012
Colombia	COL	The Americas	Upper middle	30	22	17	852.8	48,653	75.1	2011
Congo	COG	Africa	Lower middle	22	16	13	202.7	5126	64.3	2013
Costa Rica	CRI	The Americas	Upper middle	27	20	15	1286.5	4857	79.6	2014
Côte d’Ivoire	CIV	Africa	Lower middle	39	32	25	189.6	23,696	54.6	2014
Croatia	HRV	Europe	High	48	41	31	1656.4	4213	78.3	2010
Cuba	CUB	The Americas	Upper middle	31	26	20	2478.8	11,476	79	2012
Czech Republic	CZE	Europe	High	65	60	49	2469.9	10,611	79.2	2012
Democratic Peoples Republic of Korea	PRK	South-East Asia	Low	20	13	11		25,369	71.9	2012
Democratic Republic of Congo	COD	Africa	Low	22	16	13	34	78,736	60.5	2010
Djibouti	DJI	Eastern Mediterranean	Lower middle	19	13	10	146.7	942	63.8	2007
Dominica	DMA	The Americas	Upper middle	27	20	16	585.7	74		2007
Dominican Republic	DOM	The Americas	Upper middle	31	24	19	873.1	10,649	73.5	2015
Ecuador	ECU	The Americas	Upper middle	28	19	15	980.2	16,385	76.5	2013
Egypt	EGY	Eastern Mediterranean	Lower middle	27	19	15	495.2	95,689	70.5	2012
El Salvador	SLV	The Americas	Lower middle	32	25	19	578.5	6345	73.7	2009
Eritrea	ERI	Africa	Low	23	16	15	56.2	4955	65	2010
Estonia	EST	Europe	High	48	45	36	1886.8	1312	77.8	2012
Ethiopia	ETH	Africa	Low	50	40	32	65.6	102,403	65.5	2014
Fiji	FJI	Western Pacific	Upper middle	21	15	12	331.4	899	69.9	2015
Gambia	GMB	Africa	Low	15	9	9	114.1	2039	61.9	2001
Georgia	GEO	Europe	Lower middle	21	14	10	717.7	3925	72.6	2007
Ghana	GHA	Africa	Lower middle	26	20	18	249.3	28,207	63.4	2010
Grenada	GRD	The Americas	Upper middle	28	21	17	677.5	107	73.4	2007
Guinea	GIN	Africa	Low	33	26	23	57.2	12,396	59.8	2012
Guyana	GUY	The Americas	Upper middle	25	19	16	336.1	773	66.2	2010
Haiti	HTI	The Americas	Low	24	17	13	120.1	10,847	63.5	2012
Honduras	HND	The Americas	Lower middle	29	23	18	353.4	9113	75.2	2009
India	IND	South-East Asia	Lower middle	30	22	17	237.7	1,324,171	68.8	2015
Indonesia	IDN	South-East Asia	Lower middle	23	16	12	369.3	261,115	69.3	2011
Iran (Islamic Republic of)	IRN	Eastern Mediterranean	Upper middle	47	41	29	1261.7	80,277	75.7	2014
Iraq	IRQ	Eastern Mediterranean	Upper middle	46	40	33	481	37,203	69.8	2010
Jamaica	JAM	The Americas	Upper middle	40	34	26	511.4	2881	76	2012
Jordan	JOR	Eastern Mediterranean	Upper middle	51	46	37	568.1	9456	74.3	2011
Kenya	KEN	Africa	Lower middle	26	21	15	157.2	48,462	66.7	2016
Kiribati	KIR	Western Pacific	Lower middle	22	15	13	151.8	114	66.1	2009
Kyrgyzstan	KGZ	Europe	Lower middle	36	29	21	286.6	5956	71.4	2009
Latvia	LVA	Europe	High	46	43	36	1429.3	1971	75	2012
Lebanon	LBN	Eastern Mediterranean	Upper middle	30	24	18	1117.3	6007	76.3	2014
Lesotho	LSO	Africa	Lower middle	20	13	11	251.1	2204	52.9	2005
Liberia	LBR	Africa	Low	16	11	9	127.8	4614	62.9	2011
Lithuania	LTU	Europe	High	49	46	40	1874.6	2908	75	2012
Madagascar	MDG	Africa	Low	16	10	10	76.7	24,895	66.1	2008
Malawi	MWI	Africa	Low	25	18	16	108.2	18,092	64.2	2015
Malaysia	MYS	Western Pacific	Upper middle	25	18	15	1063.9	31,187	75.3	2014
Maldives	MDV	South-East Asia	Upper middle	43	36	27	1513.9	428	78.4	2011
Mali	MLI	Africa	Low	24	17	12	118.5	17,995	58	2012
Malta	MLT	Europe	High	51	44	38	3470.9	429	81.5	2008
Marshall Islands	MHL	Western Pacific	Upper middle	21	16	13	862.8	53		2007
Mauritania	MRT	Africa	Lower middle	19	13	11	177.1	4301	63.9	2008
Mexico	MEX	The Americas	Upper middle	50	44	31	1008.7	127,540	76.6	2011
Mongolia	MNG	South-East Asia	Lower middle	24	17	14	469.6	3027	69.8	2009
Montenegro	MNE	Europe	Upper middle	41	36	25	957	629	76.8	2011
Morocco	MAR	Eastern Mediterranean	Lower middle	31	24	18	435.3	35,277	76	2012
Mozambique	MOZ	Africa	Low	21	15	12	63.7	28,829	60.1	2016
Myanmar (Burma)	MMR	South-East Asia	Lower middle	30	22	18	267.2	52,885	66.8	2010
Namibia	NAM	Africa	Upper middle	27	20	16	942.5	2480	63.7	2016
Nepal	NPL	South-East Asia	Low	22	15	11	150.6	28,983	70.2	2011
Nicaragua	NIC	The Americas	Lower middle	26	19	16	406	6150	75.5	2011
Nigeria	NGA	Africa	Lower middle	21	15	13	215.2	185,990	55.2	2010
Oman	OMN	Eastern Mediterranean	High	38	32	23	1635.9	4425	77	2009
Pakistan	PAK	Eastern Mediterranean	Lower middle	26	19	14	134.4	193,203	66.5	2016
Papua New Guinea	PNG	Western Pacific	Lower middle	20	13	11	98.6	8085	65.9	2012
Paraguay	PRY	The Americas	Upper middle	28	21	16	724.3	6725	74.2	2009
Peru	PER	The Americas	Upper middle	33	25	18	671	31,774	75.9	2012
Philippines	PHL	Western Pacific	Lower middle	46	39	31	322.8	103,320	69.3	2008
Poland	POL	Europe	High	47	45	34	1704.2	38,224	77.8	2017
Portugal	PRT	Europe	High	71	67	50	2661.4	10,372	81.5	2011
Republic of Moldova	MDA	Europe	Lower middle	37	31	24	515.3	4060	71.5	2011
Romania	ROU	Europe	Upper middle	55	49	41	1090.4	19,778	75.2	2012
Russian Federation	RUS	Europe	Upper middle	36	31	21	1414	143,965	71.9	2014
Rwanda	RWA	Africa	Low	21	14	12	143.2	11,918	68	2010
Saint Lucia	LCA	The Americas	Upper middle	28	21	17	681.4	178	75.6	2007
Saint Vincent and the Grenadines	VCT	The Americas	Upper middle	26	18	14	469.5	110	72	2010
Senegal	SEN	Africa	Low	24	17	13	97.1	195	75.1	2013
Serbia	SRB	Europe	Upper middle	50	42	31	1323.7	8820	76.3	2010
Seychelles	SYC	Africa	High	24	18	13	867.3	94	73.3	2010
Slovakia	SVK	Europe	High	73	65	54	2062	5444	77.4	2012
Slovenia	SVN	Europe	High	56	54	41	2733.8	2078	80.9	2017
Solomon Islands	SLB	Western Pacific	Lower middle	23	17	13	173	599	71.1	2017
Somalia	SOM	Africa	Low	8	6	7		14,318	55.4	2006
South Africa	ZAF	Africa	Upper middle	21	14	12	1086.4	56,015	63.6	2014
Sri Lanka	LKA	South-East Asia	Lower middle	22	18	11	353.1	20,798	75.3	2013
Sudan	SDN	Africa	Lower middle	35	28	22	277	39,579	65.1	2014
Suriname	SUR	The Americas	Upper middle	25	18	14	1016.9	558	71.8	2014
Sweden	SWE	Europe	High	31	29	16	5298.6	9838	82.4	2016
Syrian Arab Republic	SYR	Eastern Mediterranean	Low	64	57	46		18,430	63.8	2008
Tajikistan	TJK	Europe	Low	26	19	15	192.7	8735	70.8	2009
Thailand	THA	South-East Asia	Upper middle	34	28	21	610.2	68,864	75.5	2013
The former Yugoslav Republic of Macedonia	MKD	Europe	Upper middle	35	28	17	857.1	2081	75.9	2008
Timor-Leste	TLS	South-East Asia	Lower middle	24	17	13	141.3	1269	68.6	2015
Togo	TGO	Africa	Low	28	21	15	95.6	7606	60.6	2012
Tonga	TON	Western Pacific	Upper middle	23	16	12	323.8	107	73.4	2007
Trinidad & Tobago	TTO	The Americas	High	44	39	32	2204.1	1365	71.8	2010
Tunisia	TUN	Eastern Mediterranean	Lower middle	58	52	43	774.1	11,403	76	2012
Uganda	UGA	Africa	Low	26	19	16	138.5	41,488	62.5	2012
Ukraine	UKR	Europe	Lower middle	20	13	10	469.4	44,439	72.5	2009
United Republic of Tanzania	TZA	Africa	Low	36	28	21	96.5	55,572	63.9	2013
Uruguay	URY	The Americas	High	41	35	28	1747.8	3444	77.1	2011
Vanuatu	VUT	Western Pacific	Lower middle	18	12	9	106.1	270	72	2006
Venezuela (Bolivarian Republic of)	VEN	The Americas	Upper middle	25	19	14	579.4	31,568	74.1	2004
Viet Nam	VNM	Western Pacific	Lower middle	60	53	43	334.3	94,569	76.3	2008
Yemen	YEM	Eastern Mediterranean	Low	21	14	11	144.5	27,584	65.3	2009
Zambia	ZMB	Africa	Lower middle	26	19	17	203	16,591	62.3	2013
Zimbabwe	ZWE	Africa	Low	26	18	14	182.3	16,150	61.4	2011

Geographic region was retrieved from the World Health Organization; Income group was retrieved from the World Bank; Population size, health expenditure and life expectancy were retrieved from the Global Health Observatory; ISO: The International Organization for Standardization- ISO-3166 Alpha-3 country code (Source: https://www.iso.org/iso-3166-country-codes.html)

## Data extraction

Using the identified guidelines for ischemic heart disease, cerebrovascular disease and hypertensive heart disease, medications used to treat these conditions were abstracted. If a guideline indicated a therapeutic class of medicines, that class was fully expanded to include all medicines because medicines within the same chemical subgroup may be considered therapeutically similar. The WHO Model List recognizes interchangeability of certain medicines on their list for others within the same therapeutic class [16]. Using this principle, 4th level Anatomical Therapeutic Chemical Classification (ATC) codes [18] were used to guide which medicines are in the same therapeutic class. If a therapeutic class was mentioned and specific alternatives were stated, only those medicines were included (no therapeutic class expansion was done).

Medicines listed on the WHO Model List or those from guidelines appearing on the WHO Model List (in a form that is usable for the conditions or cause), with a square box symbol, were fully expanded based on the 4th level, chemical subgroup of the ATC code to include all medicines within that therapeutic class. If the medicine is not denoted with a square box it was not expanded. If specific medicines considered equivalent were stated, only those medicines were included. A medicine coverage score was created by summing the number of medicines on a country’s NEML that were also listed on our list of medicines used to treat each HAQ cause.

### Data analysis

Data was analyzed using IBM SPSS Statistics version 26 (IBM Corp., 2018), and a p-value ≤ 0.05 was considered significant. An ordinary least squares linear regression model was used to test the hypothesis that there would be a positive relationship between listing medicines (medicine coverage score) and HAQ scores. HAQ score was used as the dependent variable and the previously calculated medicine coverage score was used as the independent variable. Linear regression results are reported for both unadjusted and adjusted with health expenditure, population, life expectancy and prevalence as covariates.

## Results

In total, 131 countries were included in the analysis having both a NEML and HAQ score (Table 1). WHO regions represented by countries were the Eastern Mediterranean (14 countries), Europe (26 countries), Africa (38 countries), the Americas (29 countries), South-East Asia (13 countries) and the Western Pacific (11 countries) [17]. Using the World Bank categorization, included countries represented a range of income levels with 28 low income countries, 40 lower-middle income countries, 43 upper middle countries and 20 high income countries [19]. Three countries (Democratic Peoples Republic of Korea, Somalia and Syrian Arab Republic) were excluded from the regression analysis because they were missing values for healthcare expenditure.

**Fig. 1:**
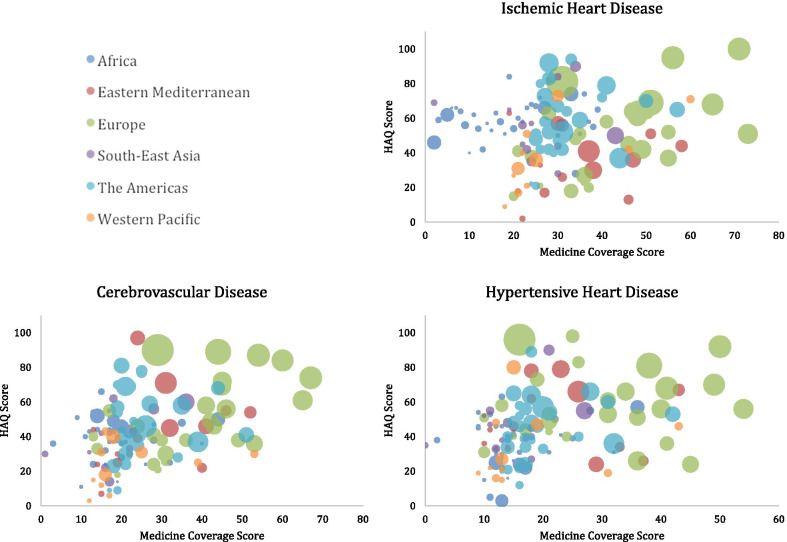
Medicine Coverage Scores. Bubble size represents health expenditure for 2015 (per capita based on purchasing power parity in international dollars); HAQ: healthcare access and quality score

The total number of medicines identified through guideline searches for each cause was 103 medicines for ischemic heart disease, 96 medicines for cerebrovascular disease and 73 medicines for hypertensive heart disease (see Additional file 1 for list of medicines). Figure 1 graphs the association between medicine coverage score and HAQ score, with health expenditure represented by bubble size.

## Ischemic heart disease

For ischemic heart disease, medicine coverage scores ranged from 2 to 73 (median: 28, IQR: 23 to 37). Results of the unadjusted linear regression model show that listing ischemic heart disease medicines only explained 0.5% of the variability in the HAQ scores across countries. After adjusting for population size, health expenditure, life expectancy and prevalence, approximately 18% of differences in the HAQ score for ischemic heart disease were explained. In the adjusted regression, there was no association between medicine coverage score and HAQ score for ischemic heart disease (*p* = 0.252), however other variables showed an association with HAQ score. Health expenditure was associated with a 0.011 point increase in HAQ score for each additional per capita dollar (*p* < 0.001) and prevalence of ischemic heart disease was associated with a 0.007 point decrease in HAQ score for each additional 100, 000 people diagnosed with ischemic heart disease (*p* < 0.001) (Table 2).

**Table 2:** Ischemic Heart Disease: Medicine Coverage Score

	Variable	B	95% CI lower bound	95% CI upper bound	Beta	P value	Pearson correlation
Unadjusted	Medicine coverage score	0.109	− 0.164	0.382	0.69	0.43	0.069
Adjusted	Medicine coverage score	0.194	− 0.14	0.528	0.123	0.252	0.108
	Health expenditure	0.011	0.005	0.017	0.467	< 0.001	0.232
	Population	− 1.056E−7	0	0	0.001	0.991	− 0.008
	Life expectancy	0.058	− 0.636	0.752	0.02	0.869	0.093
	Prevalence	− 0.007	− 0.01	− 0.004	− 0.49	< 0.001	− 0.108

R^2^_unadjusted_ = 0.005 (F = 0.626, (df 130), p = 0.43). R^2^_adjusted_ = 0.176 (F = 5.131, (df 125), p < 0.001)

B, unstandardized coefficient; Beta, standardized coefficient; CI, confidence interval

## Cerebrovascular disease

For cerebrovascular disease, medicine coverage scores ranged from 1 to 67 (median: 21, IQR 17–31). Results of the unadjusted linear regression model show that listing cerebrovascular disease medicines explained approximately 15% of the variation in the HAQ scores. After adjusting for covariates approximately 44% of differences in the HAQ score for cerebrovascular disease were explained. In the unadjusted regression, there was an association between medicine coverage score and HAQ score for cerebrovascular disease (*p* < 0.001), however the relationship was not present when covariates were included (*p* = 0.194). Similar to ischemic heart disease, other variables in the adjusted analysis showed a significant association with HAQ scores. Health expenditure was associated with a 0.014 point increase in HAQ score for each additional per capita dollar (*p* < 0.001), life expectancy was associated with a 0.557 point increase with each additional year of life (*p* = 0.042) and prevalence of cerebrovascular disease was associated with a 0.008 point decrease in HAQ score for each additional 100, 000 people diagnosed with cerebrovascular disease (*p* = 0.001) (Table 3).

**Table 3:** Cerebrovascular disease: medicine coverage score

	Variable	B	95% CI lower bound	95% CI upper bound	Beta	P-value	Pearson correlation
Unadjusted	Medicine coverage score	0.565	0.333	0.796	0.391	< 0.001	0.391
Adjusted	Medicine coverage score	0.173	− 0.089	0.435	0.117	0.194	0.393
	Health expenditure	0.014	0.009	0.018	0.587	< 0.001	0.609
	Population	− 3.977E−06	0	0	− 0.037	0.596	− 0.092
	Life expectancy	0.557	0.02	1.095	0.2	0.042	0.476
	Prevalence	− 0.008	− 0.013	− 0.004	− 0.319	0.001	0.185

R^2^_unadjusted_ = 0.153 (F = 23.225, (df 130), p < 0.001). R^2^_adjusted_ = 0.443 (F = 19.071, (df 125), p < 0.001)

B, unstandardized coefficient; Beta, standardized coefficient; CI, confidence interval

## Hypertensive heart disease

For hypertensive heart disease, medicine coverage scores ranged from 0 to 54 (median 17, IQR 13–25). Results of the unadjusted linear regression model show that listing hypertensive heart disease medicines explained approximately 11% of the variation in the HAQ score. Results of the adjusted analysis show that approximately 45% of differences in HAQ score were explained. Similar to cerebrovascular disease, an association between medicine coverage score and the HAQ score was observed for hypertensive heart disease (*p <* 0.001), however the multivariate relationship was not present when covariates were included (*p* = 0.209). Other variables in the adjusted analysis showed a significant association with HAQ scores. Health expenditure was associated with a 0.008 point increase in HAQ score for each additional per capita dollar (*p <* 0.001), life expectancy was associated with a 1.371 point increase with each additional year of life (*p <* 0.001) and prevalence of hypertensive heart disease was associated with a 0.044 point decrease in HAQ score for each additional 100,000 people diagnosed with hypertensive heart disease (*p <* 0.001) (Table 4).

**Table 4:** Hypertensive heart disease: medicine coverage score

	Variable	B	95% CI lower bound	95% CI upper bound	Beta	P-value	Pearson correlation
Unadjusted	Medicine coverage score	0.621	0.312	0.929	0.331	< 0.001	0.331
Adjusted	Medicine coverage score	0.204	− 0.116	0.524	0.11	0.209	0.324
	Health expenditure	0.008	0.004	0.013	0.346	< 0.001	0.533
	Population	2.073E−06	0	0	0.019	0.782	− 0.009
	Life expectancy	1.371	0.829	1.913	0.484	< 0.001	0.554
	Prevalence	− 0.044	− 0.063	− 0.026	− 0.402	< 0.001	0.084

R^2^_unadjusted_ = 0.109 (F = 15.846, (df 130), p < 0.001). R^2^_adjusted_ = 0.454 (F = 19.963, (df 125), p < 0.001)

B, unstandardized coefficient; Beta, standardized coefficient; CI, confidence interval

## Discussion

The number of medicines used to treat cerebrovascular disease and hypertensive heart disease included in national essential medicines lists was associated with amenable mortality, but the association was not present when country characteristics such as health spending were accounted for.

Our findings suggest that increases in a country’s health expenditure may improve HAQ scores for cardiovascular disease. Fullman et al., (2018) found that health spending per capita was strongly correlated with HAQ Index performance, however there was a large variation in score within similar levels of spending [4]. Government spending as a fraction of total health spending was also positively correlated with HAQ Index performance [4]. Per-capita health expenditure is inadequate to pay for basic healthcare interventions in some low-income countries [20, 21]. For the countries included in this study, 62 countries’ (of the 131 total countries; one country had no data) per-capita government expenditure on health was less than the minimum required for basic effective public-health system [20]. A modest increase in public spending, efficient resource use and an investment in prevention programs is necessary for addressing inequity in healthcare [21]. It is also possible that higher healthcare spending would allow countries to purchase a better selection of medicines which may, in turn, lead to better health outcomes or higher spending could increase the availability of essential medicines.

We suspect that barriers within the healthcare system are particularly important for cardiovascular health. Inequity exists within the implementation of cost-effective interventions and the provision of care for cardiovascular disease predominantly in low-income countries where health systems may not be adequately equipped for providing chronic disease care [21]. For example, in Kenya, cardiovascular medicines can only be prescribed by physicians, [22] however it can be difficult for patients to access physicians due to a lack of effective referral networks [23] and a shortage of physicians making it difficult to contend with the disease burden [22]. Therefore, patients may be entering the healthcare system but not receiving proper cardiovascular care.

Other factors, such as quality of care, may impact mortality from cardiovascular disease. A study of 137 low- and middle-income countries found that amenable mortality outcomes were predominantly due to poor quality healthcare (84% of cardiovascular deaths amenable to healthcare), while the remaining 16% was due to non-utilization of healthcare [24]. This study shows that cardiovascular deaths for people entering the healthcare system are predominantly driven by poor quality of care. Therefore quality of care may account for some of the observed differences in amendable mortality and this would attenuate any real relationship between medicine selection and health outcomes.

## Strengths and limitations

This was the first study we are aware of to compare NEML medications listings for cardiovascular diseases on a large scale. As a cross-sectional study, it would be inappropriate to draw causal conclusions about a relationship between medicine coverage scores and HAQ scores. Studying these associations over time may help solidify the conclusions drawn in this cross-sectional study. Applying a global medicine coverage score calculation represents a number of challenges. The score does not account for medicines that are therapeutically interchangeable within a class; theoretically, only one medicine in the class needs to be present for treatment, and the others are redundant. However, listing more than one medicine in a class can be beneficial in certain circumstances, for example in the case of drug recalls or shortages. In addition, there are no guidelines for the number of medicines needed in a class for proper coverage so we opted to include any that were listed in the country score. Although there are limitations to creating a medicine coverage score, our approach that was based on total medicines listed on NEMLs, allowed for an overall score that could be compared across many countries. The HAQ Index and GEM database both have their own limitations, which can be found in their respective articles [4, 10].

## Conclusions

The number of medicines relevant to cardiovascular disease included in NEMLs is associated with amenable cardiovascular mortality but this association is not present when accounting for country attributes such as national healthcare spending. Country attributes may influence essential medicine listing which can impact health outcomes.

## Supplementary Information

**Additional file 1**.

## Data Availability

The datasets analyzed during the current study are publicly available in the GEM database (Persaud et al. [10]) and in Fullman et al. [4]. (10.6084/m9.figshare.7814246.v1; https://www.thelancet.com/journals/lancet/article/PIIS0140-6736(18)30994-2/fulltext)

## Abbreviations

ATC: Anatomical Therapeutic Chemical Classification
GEM: Global essential medicines
HAQ: Healthcare access and quality
NCD: Non-communicable disease
NEML: National essential medicines list
WHO: World Health Organization
WHO Model List: WHO model list of essential medicines
