# Supplementary material for: Associations between essential medicines and health outcomes for cardiovascular disease
Source: BMC Cardiovasc Disord. 2021 Mar 25;21:151. doi: 10.1186/s12872-021-01955-1 (PMC7992928; doi:10.1186/s12872-021-01955-1)
Supplement: Supplementary file 1 — Additional file 1. [file 12872_2021_1955_MOESM1_ESM.docx]

# Additional file 1

*Medicines for Ischemic heart Disease*

| **Medicine Name** | **Primary ATC Code** |
| --- | --- |
| Acebutolol | C07AB04 |
| Acenocoumarol | B01AA07 |
| Acetylsalicylic acid | B01AC06 |
| Alteplase | B01AD02 |
| Amiodarone | C01BD01 |
| Amlodipine | C08CA01 |
| Atenolol | C07AB03 |
| Atorvastatin | C10AA05 |
| Benazepril | C09AA07 |
| Bendrofluazide (Bendroflumethiazide) | C03AA01 |
| Betaxolol | C07AB05 |
| Bevantolol | C07AB06 |
| Bisoprolol | C07AB07 |
| Bumetanide | C03CA02 |
| Candesartan | C09CA06 |
| Captopril | C09AA01 |
| Carvedilol | C07AG02 |
| Celiprolol | C07AB08 |
| Chlorothiazide | C03AA04 |
| Chlortalidone (Chlorthalidone) | C03BA04 |
| Cilazapril | C09AA08 |
| Cilnidipine | C08CA14 |
| Clopidogrel | B01AC04 |
| Cyclopenthiazide | C03AA07 |
| Dalteparin | B01AB04 |
| Delapril | C09AA12 |
| Digoxin | C01AA05 |
| Dopamine | C01CA04 |
| Drotrecogin alfa | B01AD10 |
| Enalapril | C09AA02 |
| Enoxaparin | B01AB05 |
| Epanolol | C07AB10 |
| Epinephrine (Adrenaline) | C01CA24 |
| Eplerenone | C03DA04 |
| Eprosartan | C09CA02 |
| Esmolol | C07AB09 |
| Ethyl biscoumacetate | B01AA08 |
| Felodipine | C08CA02 |
| Fibrinolysin | B01AD05 |
| Fluindione | B01AA12 |
| Fluvastatin | C10AA04 |
| Fosinopril | C09AA09 |
| Furosemide | C03CA01 |
| Heparin | B01AB01 |
| Hydrochlorothiazide | C03AA03 |
| Hydromorphone | N02AA03 |
| Imidapril | C09AA16 |
| Indapamide | C03BA11 |
| Irbesartan | C09CA04 |
| Isosorbide | C01DA |
| Isosorbide dinitrate | C01DA08 |
| Isosorbide mononitrate | C01DA14 |
| Isradipine | C08CA03 |
| Lacidipine | C08CA09 |
| Landiolol | C07AB14 |
| Lercanidipine | C08CA13 |
| Lidocaine (Lignocaine, Xylocaine) | C05AD01 |
| Lisinopril | C09AA03 |
| Losartan | C09CA01 |
| Lovastatin | C10AA02 |
| Manidipine | C08CA11 |
| Methylchlorothiazide | C03AA |
| Metoprolol | C07AB02 |
| Moexipril | C09AA13 |
| Morphine | N02AA01 |
| Nadroparin | B01AB06 |
| Nebivolol | C07AB12 |
| Nicardipine | C08CA04 |
| Nifedipine | C08CA05 |
| Nilvadipine | C08CA10 |
| Nimodipine | C08CA06 |
| Nisoldipine | C08CA07 |
| Nitrendipine | C08CA08 |
| Nitroglycerin (Glyceryl trinitrate) | C01DA02 |
| Nitroprusside | C02DD01 |
| Olmesartan | C09CA |
| Oxygen | V03AN01 |
| Pentaerythritol tetranitrate | C01DA05 |
| Perindopril | C09AA04 |
| Phenprocoumon | B01AA04 |
| Plasminogen activator | B01AD |
| Polythiazide | C03AA05 |
| Practolol | C07AB01 |
| Pravastatin | C10AA03 |
| Prourokinase | B01AD |
| Quinapril | C09AA06 |
| Ramipril | C09AA05 |
| Reteplase | B01AD07 |
| Rosuvastatin | C10AA07 |
| Simvastatin | C10AA01 |
| Spirapril | C09AA11 |
| Spironolactone | C03DA01 |
| Streptokinase* | B01AD01 |
| Talinolol | C07AB13 |
| Telmisartan | C09CA07 |
| Tenecteplase | B01AD11 |
| Torsemide | C03CA04 |
| Trandolapril | C09AA10 |
| Urokinase | B01AD04 |
| Valsartan | C09CA03 |
| Verapamil | C08DA01 |
| Warfarin | B01AA03 |
| Zofenopril | C09AA15 |

*An exception to the rules stated in the methods was made for streptokinase, it is not denoted with a square box on the WHO Model List however, it was expanded. Two clinicians (NP, DM) agreed that it was worth expanding because all medicines in the therapeutic class were felt to be equivalent and widely used.

*Medicines for Cerebrovascular Disease*

| **Medicine Name** | **Primary ATC Code** |
| --- | --- |
| Acebutolol | C07AB04 |
| Acenocoumarol | B01AA07 |
| Acetylsalicylic acid | B01AC06 |
| Alteplase | B01AD02 |
| Amiodarone | C01BD01 |
| Amlodipine | C08CA01 |
| Apixaban | B01AF02 |
| Atenolol | C07AB03 |
| Atorvastatin | C10AA05 |
| Benazepril | C09AA07 |
| Bendrofluazide (Bendroflumethiazide) | C03AA01 |
| Betaxolol | C07AB05 |
| Bevantolol | C07AB06 |
| Bisoprolol | C07AB07 |
| Bumetanide | C03CA02 |
| Candesartan | C09CA06 |
| Captopril | C09AA01 |
| Carvedilol | C07AG02 |
| Celiprolol | C07AB08 |
| Chlorothiazide | C03AA04 |
| Chlortalidone (Chlorthalidone) | C03BA04 |
| Cilazapril | C09AA08 |
| Cilnidipine | C08CA14 |
| Clopidogrel | B01AC04 |
| Cyclopenthiazide | C03AA07 |
| Dabigatran | B01AE07 |
| Dalteparin | B01AB04 |
| Delapril | C09AA12 |
| Digoxin | C01AA05 |
| Dipyridamole | B01AC07 |
| Drotrecogin alfa | B01AD10 |
| Enalapril | C09AA02 |
| Enoxaparin | B01AB05 |
| Epanolol | C07AB10 |
| Eplerenone | C03DA04 |
| Eprosartan | C09CA02 |
| Esmolol | C07AB09 |
| Ethyl biscoumacetate | B01AA08 |
| Felodipine | C08CA02 |
| Fibrinolysin | B01AD05 |
| Fluindione | B01AA12 |
| Fluvastatin | C10AA04 |
| Fosinopril | C09AA09 |
| Furosemide | C03CA01 |
| Heparin | B01AB01 |
| Hydrochlorothiazide | C03AA03 |
| Imidapril | C09AA16 |
| Indapamide | C03BA11 |
| Irbesartan | C09CA04 |
| Isradipine | C08CA03 |
| Lacidipine | C08CA09 |
| Landiolol | C07AB14 |
| Lercanidipine | C08CA13 |
| Lisinopril | C09AA03 |
| Losartan | C09CA01 |
| Lovastatin | C10AA02 |
| Manidipine | C08CA11 |
| Methylchlorothiazide | C03AA |
| Metoprolol | C07AB02 |
| Moexipril | C09AA13 |
| Nadroparin | B01AB06 |
| Nebivolol | C07AB12 |
| Nicardipine | C08CA04 |
| Nifedipine | C08CA05 |
| Nilvadipine | C08CA10 |
| Nimodipine | C08CA06 |
| Nisoldipine | C08CA07 |
| Nitrendipine | C08CA08 |
| Nitroprusside | C02DD01 |
| Olmesartan | C09CA |
| Perindopril | C09AA04 |
| Phenprocoumon | B01AA04 |
| Plasminogen activator | B01AD |
| Polythiazide | C03AA05 |
| Practolol | C07AB01 |
| Pravastatin | C10AA03 |
| Prourokinase | B01AD |
| Quinapril | C09AA06 |
| Ramipril | C09AA05 |
| Reteplase | B01AD07 |
| Rivaroxaban | B01AF01 |
| Rosuvastatin | C10AA07 |
| Simvastatin | C10AA01 |
| Spirapril | C09AA11 |
| Spironolactone | C03DA01 |
| Streptokinase* | B01AD01 |
| Talinolol | C07AB13 |
| Telmisartan | C09CA07 |
| Tenecteplase | B01AD11 |
| Torsemide | C03CA04 |
| Trandolapril | C09AA10 |
| Urokinase | B01AD04 |
| Valsartan | C09CA03 |
| Verapamil | C08DA01 |
| Warfarin | B01AA03 |
| Zofenopril | C09AA15 |

*An exception to the rules stated in the methods was made for streptokinase, it is not denoted with a square box on the WHO Model List however, it was expanded. Two clinicians (NP, DM) agreed that it was worth expanding because all medicines in the therapeutic class were felt to be equivalent and widely used.

*Medicines for Hypertensive Heart Disease*

| **Medicine Name** | **Primary ATC Code** |
| --- | --- |
| Acebutolol | C07AB04 |
| Amlodipine | C08CA01 |
| Atenolol | C07AB03 |
| Atorvastatin | C10AA05 |
| Benazepril | C09AA07 |
| Bendrofluazide (Bendroflumethiazide) | C03AA01 |
| Betaxolol | C07AB05 |
| Bevantolol | C07AB06 |
| Bisoprolol | C07AB07 |
| Bumetanide | C03CA02 |
| Candesartan | C09CA06 |
| Captopril | C09AA01 |
| Carvedilol | C07AG02 |
| Celiprolol | C07AB08 |
| Chlorothiazide | C03AA04 |
| Chlortalidone (Chlorthalidone) | C03BA04 |
| Cilazapril | C09AA08 |
| Cilnidipine | C08CA14 |
| Cyclopenthiazide | C03AA07 |
| Delapril | C09AA12 |
| Digoxin | C01AA05 |
| Dopamine | C01CA04 |
| Enalapril | C09AA02 |
| Epanolol | C07AB10 |
| Eplerenone | C03DA04 |
| Eprosartan | C09CA02 |
| Esmolol | C07AB09 |
| Felodipine | C08CA02 |
| Fluvastatin | C10AA04 |
| Fosinopril | C09AA09 |
| Furosemide | C03CA01 |
| Hydralazine | C02DB02 |
| Hydrochlorothiazide | C03AA03 |
| Imidapril | C09AA16 |
| Indapamide | C03BA11 |
| Irbesartan | C09CA04 |
| Isradipine | C08CA03 |
| Lacidipine | C08CA09 |
| Landiolol | C07AB14 |
| Lercanidipine | C08CA13 |
| Lisinopril | C09AA03 |
| Losartan | C09CA01 |
| Lovastatin | C10AA02 |
| Manidipine | C08CA11 |
| Methylchlorothiazide | C03AA |
| Methyldopa | C02AB |
| Metoprolol | C07AB02 |
| Moexipril | C09AA13 |
| Nebivolol | C07AB12 |
| Nicardipine | C08CA04 |
| Nifedipine | C08CA05 |
| Nilvadipine | C08CA10 |
| Nimodipine | C08CA06 |
| Nisoldipine | C08CA07 |
| Nitrendipine | C08CA08 |
| Nitroprusside | C02DD01 |
| Olmesartan | C09CA |
| Perindopril | C09AA04 |
| Polythiazide | C03AA05 |
| Practolol | C07AB01 |
| Pravastatin | C10AA03 |
| Quinapril | C09AA06 |
| Ramipril | C09AA05 |
| Rosuvastatin | C10AA07 |
| Simvastatin | C10AA01 |
| Spirapril | C09AA11 |
| Spironolactone | C03DA01 |
| Talinolol | C07AB13 |
| Telmisartan | C09CA07 |
| Torsemide | C03CA04 |
| Trandolapril | C09AA10 |
| Valsartan | C09CA03 |
| Zofenopril | C09AA15 |
